# Foreground-Background Segmentation Revealed during Natural Image Viewing

**DOI:** 10.1523/ENEURO.0075-18.2018

**Published:** 2018-09-19

**Authors:** Paolo Papale, Andrea Leo, Luca Cecchetti, Giacomo Handjaras, Kendrick N. Kay, Pietro Pietrini, Emiliano Ricciardi

**Affiliations:** 1 Molecular Mind Lab, IMT School for Advanced Studies Lucca, Lucca, 55100 Italy; 2 Center for Magnetic Resonance Research, Department of Radiology, University of Minnesota, Twin Cities, Minneapolis, MN, 55455

**Keywords:** figure-ground, fMRI, human, natural scenes, visual cortex, visual perception

## Abstract

One of the major challenges in visual neuroscience is represented by foreground-background segmentation. Data from nonhuman primates show that segmentation leads to two distinct, but associated processes: the enhancement of neural activity during figure processing (i.e., foreground enhancement) and the suppression of background-related activity (i.e., background suppression). To study foreground-background segmentation in ecological conditions, we introduce a novel method based on parametric modulation of low-level image properties followed by application of simple computational image-processing models. By correlating the outcome of this procedure with human fMRI activity, measured during passive viewing of 334 natural images, we produced easily interpretable “correlation images” from visual populations. Results show evidence of foreground enhancement in all tested regions, from V1 to lateral occipital complex (LOC), while background suppression occurs in V4 and LOC only. Correlation images derived from V4 and LOC revealed a preserved spatial resolution of foreground textures, indicating a richer representation of the salient part of natural images, rather than a simplistic model of object shape. Our results indicate that scene segmentation occurs during natural viewing, even when individuals are not required to perform any particular task.

## Significance Statement

Foreground-background segmentation has been considered critical to form discrete object representations from continuous sensory percepts. We developed a pre-filtering approach which overcame typical limitations in modeling brain responses to complex stimuli and could be generalized to related processes. Our findings provide novel support to the hypothesis that foreground-background segmentation of natural scenes occurs during passive perception, sustained by the distributed activity of multiple areas across the visual processing stream. Specifically, while foreground information is enhanced along the entire visual pathway, V4 and lateral occipital complex (LOC) show a background suppression effect, though retaining texture information from the foreground. Our observations challenge the idea that these regions of the visual system may primarily encode simple object representations based on silhouette or shape features only.

## Introduction

In the scientific journey toward a satisfying understanding of the human visual system, scene segmentation represents a central problem “for which no theoretical solution exists” (Wu et al., 2006). Segmentation into foreground and background is crucial to make sense of the surrounding visual environment, and its pivotal role as an initial step of visual content identification has long been theorized (Biederman, 1987). Indeed, according to Fowlkes et al. (2007), humans can produce consistent segmentations of natural images. However, although more recent approaches based on deep convolutional networks produced promising results (He et al., 2017), both the computational and neurophysiological processes that underlie scene segmentation are still a matter of debate.

To date, numerous studies found evidence of texture segmentation and figure-ground organization in the early visual cortex of nonhuman primates (Lamme, 1995; Lee et al., 1998; Poort et al., 2012; Self et al., 2013) and humans (Kastner et al., 2000; Scholte et al., 2008; Kok and de Lange, 2014). It has been showed that the identification of salient visual attributes arises from a region-filling mechanism that targets neural populations mapping relevant points in space (Roelfsema, 2006). In particular, a recent study on monkeys attending artificial stimuli revealed an early enhancement of V1 and V4 neurons when their receptive fields covered the foreground and a later response suppression when their receptive fields were located in the stimulus background (Poort et al., 2016), extending results from a previous study (Lamme et al., 1999). Thus, the primate brain groups together image elements which belong to the figure, showing an enhanced activity for the foreground and a concurrent suppression of the background.

However, from an experimental viewpoint, the role of figure-ground segmentation has primarily been demonstrated by means of non-ecological stimuli (e.g., binary figures, random dots, oriented line segments and textures). It should be noted that previous reports demonstrated how models of brain responses to artificial stimuli are suboptimal in predicting responses to natural images (David et al., 2004; Felsen and Dan, 2005). Although two recent studies investigated border-ownership in monkeys with both artificial and natural stimuli (Hesse and Tsao, 2016; Williford and von der Heydt, 2016), a proof of the occurrence of foreground-background segmentation in the human brain during visual processing of naturalistic stimuli (e.g., natural images and movies) is still lacking. This pushes toward the development of novel methods specifically designed for testing segmentation in ecological conditions.

In light of this, we investigated foreground enhancement and background suppression, as specific processes involved in scene segmentation during passive viewing of natural images. We used fMRI data, previously published by Kay et al. (2008), to study brain activity patterns from seven visual regions of interest (ROIs): V1, V2, V3, V3A, V3B, V4, and lateral occipital complex (LOC) in response to 334 natural images, whose “ground-truth” segmented counterparts have been included in the Berkeley Segmentation Dataset (BSD; Arbeláez et al., 2011).

**Figure 1.**
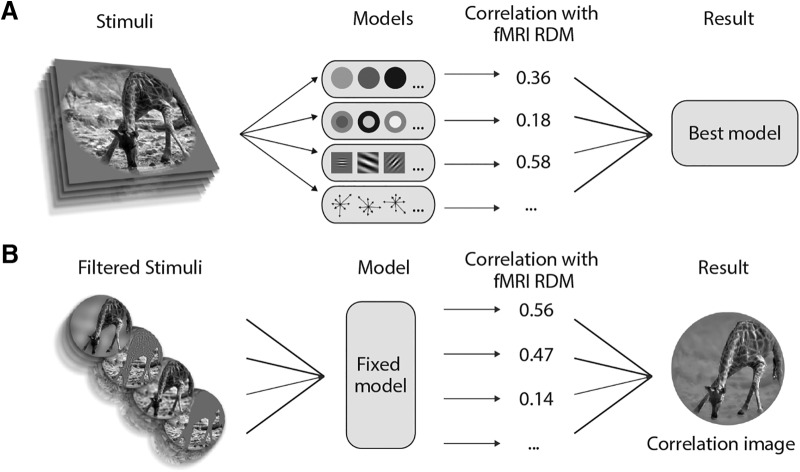
Comparing the standard modeling approach and the pre-filtering modeling approach. ***A***, In the standard modeling pipeline, different models are compared. After extracting features from the stimuli, competing feature vectors can be used to predict brain activity in an encoding procedure, whereas their dissimilarities can be used in a RSA. Finally, the model that better predicts brain responses is discussed. ***B***, In our pre-filtering modeling approach, different filtered versions of the original stimuli are compared. Various biologically plausible filtering procedures are applied to the stimuli before compute a unique feature space according to a given fixed and easily interpretable model. In our approach, a single model is employed and the step showing the highest correlation with brain activity (or representational geometry) of each filtering procedure is used to build a *post hoc* correlation image. While the standard modeling approach is theoretically more advantageous, as its output is a fully computable model of brain activity, it cannot be applied when reliable explicit models of perceptual processes do not exist yet, as in the case of scene segmentation. Alternative attempts to reconstruct visual stimuli from brain activity have been previously reported using multivariate techniques (Stanley et al., 1999; Thirion et al., 2006; Miyawaki et al., 2008; Nishimoto et al., 2011).

To this aim, we developed a novel pre-filtering modeling approach to study brain responses to complex, natural images without relying on explicit models of scene segmentation and adopting a validated and biologically plausible description of activity in visual cortices. Our method is similar to other approaches where explicit computations are performed on representational features, rather than on the original stimuli (Naselaris et al., 2011). For instance, these methods have been recently used to investigate semantic representation (Huth et al., 2012; Handjaras et al., 2017) or boundary and surface-related features (Lescroart et al., 2016). However, as opposed to the standard modeling framework, according to which alternative models are computed from the stimuli to predict brain responses, here, low-level features of the stimuli are parametrically modulated and simple descriptors of each filtered image (i.e., edges position, size and orientation) are aggregated in a fixed model (Fig. 1). The correspondence between the fixed model and fMRI representational geometry related to intact images, was then evaluated using representational similarity analysis (RSA; Kriegeskorte et al., 2008). Notably, this approach can also be exploited to obtain highly informative “correlation images” representing the putative computations of different brain regions and may be generalized to investigate different phenomena in visual neuroscience.

## Materials and Methods

**Figure 2.**
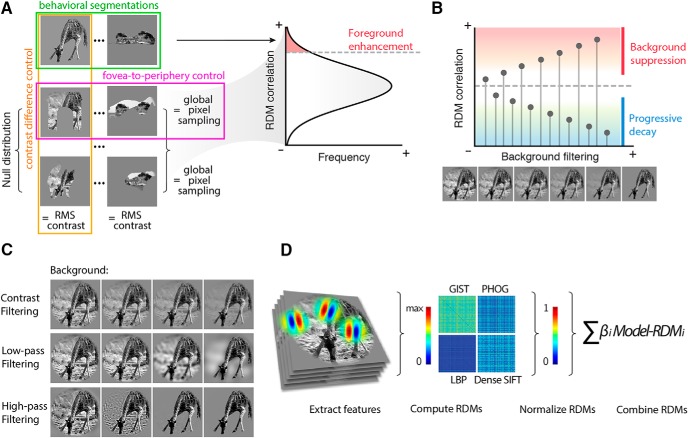
Analytical pipeline. ***A***, Foreground enhancement test: the set of segmented stimuli is tested against a null distribution of 1000 permutations. Each permutation is built by randomly shuffling the 334 behavioral foreground masks and matching the RMS contrast of the behaviorally segmented counterpart. This analysis controls for size, location, and contrast of the foreground when testing whether behavioral segmentations explain each ROI RDM better than chance. ***B***, Background suppression test: the correlation between brain RDMs and each step of the background filtering procedure is tested against the correlation determined by the intact stimuli. While information is filtered out, correlation can increase or decrease, depending on the sensitivity for background related information in each ROI. A progressive decay indicates that a region actually processes the background, while a significant increase suggests that background is suppressed. ***C***, Filtering steps for the contrast or spatial frequencies filtering. ***D***, From left to right: features for each model were extracted from the stimuli; the dissimilarity (1-Pearson’s *r*) between each stimulus pair was computed and aggregated in four RDMs; the obtained RDMs were normalized in a 0–1 range; finally, the four RDMs were linearly combined in the fixed model, which was then correlated to the fMRI RDM obtained from each ROI.

To assess differences between cortical processes involved in foreground-background segmentation, we employed a low-level description of images (edge position, size and orientation), defined by a weighted sum of the representational dissimilarity matrices (RDMs) of four well-known computational models (Fig. 2*D*). These models are based on simple features (edge position, size and orientation), whose physiologic counterparts are well known (Marr, 1982). The model was kept constant while the images were parametrically filtered and iteratively correlated with representational measures of brain activity through RSA. For each ROI, this pre-filtering modeling approach led to a pictorial and easily interpretable representation of the optimal features (contrast and spatial frequencies) of foreground and background of natural images (i.e., correlation images). The analytical pipeline is schematized in Figure 2.

### Stimuli and behavioral segmentation of foreground and background

We selected from the 1870 images used by Kay et al. (2008) a subsample of 334 pictorial stimuli which are also represented in the BSD 500 (Arbeláez et al., 2011). For each BSD image, five to seven subjects manually performed an individual ground-truth segmentation, which is provided by the authors of the dataset (http://www.eecs.berkeley.edu/Research/Projects/CS/vision/grouping/resources.html ). Although figure-ground judgment is rather stable across subjects (Fowlkes et al., 2007), we selected the largest patch, manually labeled as foreground, among the behavioral segmentations, to build a foreground binary mask. For each image, this mask was then down-sampled and applied to the original stimulus to isolate the foreground and the background pixels (Kay et al., 2008).

### fMRI data

The fMRI data used in this study are publicly available at http://crcns.org/data-sets/vc/vim-1 (Kay et al., 2011). Two subjects (males, age: 33 and 25) were acquired using the following MRI parameters: 4T INOVA MR, matrix size 64 × 64, TR 1 s, TE 28 ms, flip angle 20°, spatial resolution 2 × 2 × 2.5 mm^3^. For each subject five scanning sessions (seven runs each) were performed on five separate days. The stimuli were 1870 greyscale natural images with diameter 20° (500 pixels), embedded in a gray background, and were presented for 1 s, flickering at 5 Hz, with an ISI of 3 s. Subjects were asked to fixate a central white square of 0.2° (4 pixels). Seven visual ROIs (V1, V2, V3, V3A, V3B, V4, and LOC) were defined and brain activity patterns related to stimulus presentation was extracted from these regions. For additional details on pre-processing, retinotopic mapping and ROIs localization, refer to Kay et al. (2008).

### Computational models

In accordance with a previous fMRI study that, to the best of our knowledge, has tested the highest number of computational models, we selected four untrained models: two showing highest correlations with brain activity patterns in early visual areas, and the others, showing highest correlations with LOC (Khaligh-Razavi and Kriegeskorte, 2014). All these models are based on biologically inspired features, such as Gabor filters and image gradient and comprise: GIST (Oliva and Torralba, 2001), Dense SIFT (Lazebnik et al., 2006), Pyramid Histograms of Gradients (PHOG; Bosch et al., 2007), and Local Binary Patterns (LBP; Ojala et al., 2001). For an exhaustive description of the four models, and links to MATLAB codes, see Khaligh-Razavi (2014) and Khaligh-Razavi and Kriegeskorte (2014). Our model choice was also motivated by the fact that the stimuli were grayscale and had a fixed circular aperture. Thus, we excluded descriptions based on color or silhouette information, as well as pretrained convolutional neural networks which are biased toward the global shape of the image (Kubilius et al., 2016).

### RSA

For each filtered image, we collected feature vectors from the four computational models (PHOG, GIST, LBP, and Dense SIFT), and RDMs were then obtained (1 minus the Pearson correlation metric). These four RDMs were normalized in a range between 0 and 1 and combined to obtain the fixed biologically plausible model of the stimuli (for a graphical representation of the process, see Fig. 2*D*). The four model RDMs were combined through a weighted sum, based on an estimation of their correlation with the representational model of brain activity. Single subject RDMs were similarly computed using fMRI activity patterns for each of the seven ROIs, and then averaged across the two subjects. We used Spearman’s ρ to assess the correlation between the RDM from each step of the image filtering procedures and the RDM of each brain ROI. To obtain unbiased estimations of the correlation between models and fMRI, a 5-fold cross-validation procedure based on a weighted sum of the models was developed: model weights were first estimated trough linear regression on a portion (80%) of the RDMs, and the correlation with fMRI data were then computed based on the remainder of the RDMs (20%). The correlation values derived from this procedure were averaged across the five folds, to obtain a unique estimate of the similarity between image features and brain activity. This analysis was performed independently in each of the seven ROIs, and the SE for each correlation value was estimated with bootstrapping of the stimuli – 1000 iterations (Efron and Tibshirani, 1993).

**Figure 3.**
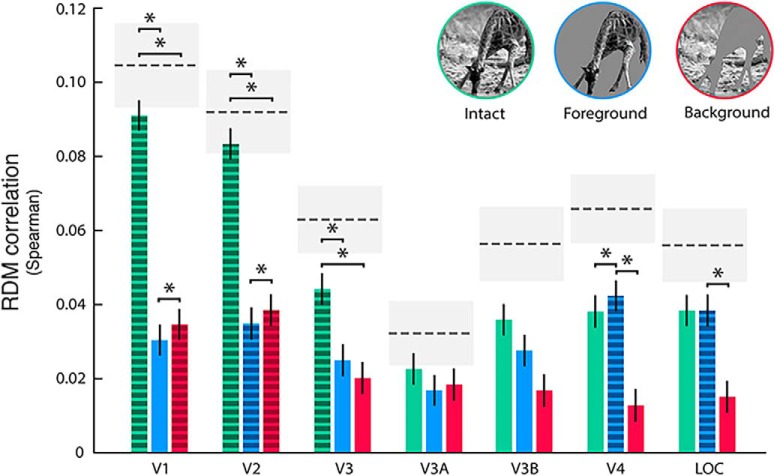
Comparison of intact and behaviorally segmented images. The graphs show the correlation between the intact (green) and segmented versions (blue: isolated foreground; red: isolated background) of the images and brain RDMs (*n* = 55611). Dashed bars stand for significant correlations as resulting from the permutation test (*p* < 0.05, Bonferroni corrected; 1000 iterations). Asterisks indicate significant differences between correlation values (*p* < 0.05, Bonferroni corrected). Error bars represent the SE estimated with bootstrapping. Dashed lines represent the SNR estimate for each ROI, while gray shaded regions indicate its SE.

In addition, as each ROI may show a distinct signal-to-noise ratio, we computed a noise estimation by correlating the brain RDMs extracted from the two subjects. This procedure allows for qualitative comparison between different ROIs and could help in estimate how well each model explains fMRI RDMs given the noise in the data. 

### Foreground enhancement testing

A permutation test was performed to statistically assess the enhancement of the information retained in the behavioral segmented foreground. In this test both the “fovea-to-periphery” bias that characterizes natural images, and possible differences in contrast between foreground and background were controlled (Fig. 2*A*). For each iteration, the 334 foreground masks were shuffled and a random foreground segmentation was associated to each stimulus. The root mean square (RMS) contrast of each obtained segmented image was matched to that of the behaviorally segmented counterpart. Of note, this set of randomly-segmented images had the same distribution of masked portions of the visual field as the one from the behavioral segmentation, so the same amount of information was isolated at each permutation step. This procedure was repeated 1000 times, to build a null distribution of alternative segmentations: four examples of random segmentation are shown in Figure 2*A*. For each permutation step, features were extracted from each randomly segmented image and RSA was performed using the procedure described above.

### Parametric filtering procedures

To investigate differential processing of foreground and background in the visual system, we employed three different filtering procedures (contrast; through alpha channel modulation; low- and high-pass filtering of spatial frequencies) applied parametrically (four steps each) to the foreground or the background. For each filtering procedure, the four manipulated images are represented in Figure 2*C*. For low- and high-pass filtering, we employed a Butterworth filter (5th order), linearly sampling from a log-transformed distribution of frequencies ranging from 0.05 to 25 cycle per degree, while keeping the RMS contrast fixed.

### Background suppression testing

To test background suppression, we performed a two-tailed permutation test. In each ROI, we computed the difference between the correlation of the intact version of the stimuli and each step of the background filtering procedures (Fig. 2*B*). Afterward, a permutation test (10,000 iterations) was performed by random sampling two groups from the bootstrap distributions, obtaining a null distribution of correlation differences. Reported results are Bonferroni corrected (for the 13 comparisons in each ROI).

### Correlation images

For each ROI, the effects of the filtering procedures were combined, to build correlation images. To this aim we used the filtering step with the highest correlation between the fixed model and RDMs from fMRI data, for foreground and background respectively. In detail, we averaged the best images for the low- and high-pass filters and multiplied each pixel for the preferred alpha-channel value (contrast).

### Significance testing

To assess the statistical significance of the correlations obtained with RSA in all the above-mentioned filtering procedures, we built a robust ROI-specific permutation test (1000 iterations), by randomly sampling voxels of the occipital lobe not located in any of the seven ROIs. We labeled these voxels as “control-voxels.” This procedure has the advantage to be resilient to biases in fMRI data (Schreiber and Krekelberg, 2013), instead of simply taking into account the distribution of the RDM values, as in Khaligh-Razavi and Kriegeskorte (2014). In addition, the procedure that we developed is also useful to control for the effects related to number of voxels and to the signal-to-noise ratio of each ROI.

First, for each ROI we computed the SE of the ROI-specific noise estimation with bootstrap resampling of the stimuli (1000 iterations). Second, a number of control voxels equal to the number of voxels was randomly selected within each ROI, and the activity of these control voxels in response to the stimuli were used to build a null RDM. Third, the correlation between the null RDMs of the two subjects was computed. However, since we aimed at matching the signal-to-noise ratio of the null distribution to that of each ROI, the null RDM was counted as a valid permutation only if the single subject RDMs correlated to each other within a specific range (i.e., ROI-specific noise estimation ± SE). Finally, for each step of the filtering procedures, each of the 1000 ROI-specific null RDMs were correlated with the fixed model RDM to obtain a null distribution of 1000 ρ values. A one-tailed rank test was used to assess the significance of the ρ of the fixed model with brain RDMs. For each ROI, we controlled for multiple comparisons (27 tests), through Bonferroni correction.

### Code accessibility

All analyses have been implemented in MATLAB (MathWorks Inc.) using in-house developed code (available at https://bit.ly/2rC27hY). All code is also available as Extended Data 1.

10.1523/ENEURO.0075-18.2018.ed1 Extended Data 1 All the code and data of the present work are publicly available. Download Extended Data 1, ZIP file.

## Results

Foreground enhancement and background suppression can be tested in ecological conditions following a simple argument: when attempting to predict brain activity of a visual ROI with a specific model, the goodness-of-fit depends on the model inputs, e.g., the spatial information provided. Thus, the correlation between filtered images and fMRI representational patterns evoked by their intact counterpart can be used to verify specific hypotheses on visual processing (Fig. 2). In this study, we posit that evidence of preferential processing (i.e., enhancement) should depend on the shape of the foreground instead of the size, the location or the contrast of the segmented region processed through the model. In this regard, a random sampling procedure of foreground segmentations across stimuli would offer a proper choice to account for all these aspects, ultimately testing whether behavioral segmentations provide a better prior for enhancement. On the other hand, background filtering can lead either to a decay or an increase in correlation with brain representational patterns. The former indicates that background-related information is, at least to some extent, processed, whereas the latter denotes that background information is suppressed, since embedding it in the model is not different from adding noise.

### Comparison of intact and behaviorally segmented images

The correlation between RDMs computed using the fMRI patterns from each of the seven visual ROIs and three descriptions of the stimuli (intact, isolated background, and isolated foreground) were tested (Fig. 3; Table 1). Results show significant correlations (*p* < 0.05 Bonferroni corrected) between the intact description of images and fMRI RDMs in V1, V2, and V3. The segmented foreground RDM shows a significant correlation in V2, V4, and LOC, while the segmented background achieves significant correlations in V1 and V2 only. Of note, the correlation yielded by one of the descriptions approaches the ROI-specific SNR estimation (i.e., the maximum reachable correlation given the noise of the data), thus confirming the validity of the fixed model employed (Wu et al., 2006).

**Table 1.** Comparison of intact and behaviorally segmented images

ROI	Intact		Foreground		Background	
	Spearman’s ρ	*p* value	Spearman’s ρ	*p* value	Spearman’s ρ	*p* value
V1	0.091 ± 0.008	<0.001^*^	0.03 ± 0.008	0.006	0.035 ± 0.008	<0.001^*^
V2	0.084 ± 0.005	<0.001^*^	0.035 ± 0.007	<0.001^*^	0.039 ± 0.004	<0.001^*^
V3	0.044 ± 0.007	<0.001^*^	0.025 ± 0.008	0.08	0.02 ± 0.007	0.29
V3A	0.023 ± 0.004	0.34	0.017 ± 0.005	0.256	0.018 ± 0.007	0.127
V3B	0.036 ± 0.009	0.017	0.028 ± 0.006	0.02	0.017 ± 0.009	0.186
V4	0.038 ± 0.015	0.027	0.043 ± 0.006	<0.001^*^	0.013 ± 0.007	0.915
LOC	0.038 ± 0.008	0.015	0.038 ± 0.012	<0.001^*^	0.015 ± 0.009	0.543

* *p* < 0.05 Bonferroni corrected.

**Table 2.** Statistical analysis

	Data structure	Type of test	Power
a	Single correlation values	Nonparametric permutation test	*p* < 0.05 Bonferroni corrected
b	Single correlation values	Nonparametric permutation test	*p* < 0.05
c	Single correlation values	Nonparametric permutation test	*p* < 0.05 Bonferroni corrected

### Foreground is enhanced in all the tested regions

We tested whether the behavioral foreground segmentation from BSD represented a better predictor of RDMs derived from fMRI activity, as compared to alternate configurations obtained by shuffling the segmentation patterns across stimuli (Fig. 2*A*). The correct foreground configuration yielded a significantly higher correlation as compared to the examples from the shuffled dataset (i.e., a null distribution obtained with a permutation test; Table 2), thus suggesting that the enhancement of foreground-related information occurs during passive perception of natural stimuli in all the tested ROIs (V1: *p* = 0.006; V2: *p* < 0.001; V3: *p* = 0.014; V3A: *p* = 0.002; V3B: *p* = 0.005; V4: *p* < 0.001; LOC: *p* < 0.001).

In addition, this analysis rules out two potential confounding effects. One related to a fovea-to-periphery bias in our image set. In fact, as already observed in literature, natural images are typically characterized by objects located at the center of the scene, see for instance the object location bias represented in Alexe et al. (2010; their Fig 3*B*). However, since the spatial distribution and number of pixels were kept constant at each permutation step, we replicated the same fovea-to-periphery bias in the null distribution. The other confound was related to potential differences in contrast between foreground and background. To account for this, in the permutation test, we matched the RMS contrast of each random segmentation to that of the ground-truth segmentation obtained from BSD. Overall, these control procedures minimize the chance that the observed enhancement is driven by location, size, or contrast of the foreground.

### Background suppression occurs in higher cortical areas

As the correlation between the background RDM and RDM derived from fMRI activity is significant in V1 and V2 only (Fig. 3), we hypothesized that background-related information is suppressed in “higher” visual cortices. Notably, Poort et al. (2016) described background suppression as a different, but associated, phenomenon with respect to foreground enhancement. Thus, to better characterize where and how background suppression occurs in humans attending to natural images, a further analysis was performed by parametrically filtering out the background of each image, varying its contrast or spatial frequencies (low- and high-pass filtering; Fig. 2*C*). As the correlation between the representational model of V3A, V3B, and those derived from intact, isolated foreground, and isolated background images is not significant (*p* > 0.05 Bonferroni corrected), these ROIs were not further investigated.

**Figure 4.**
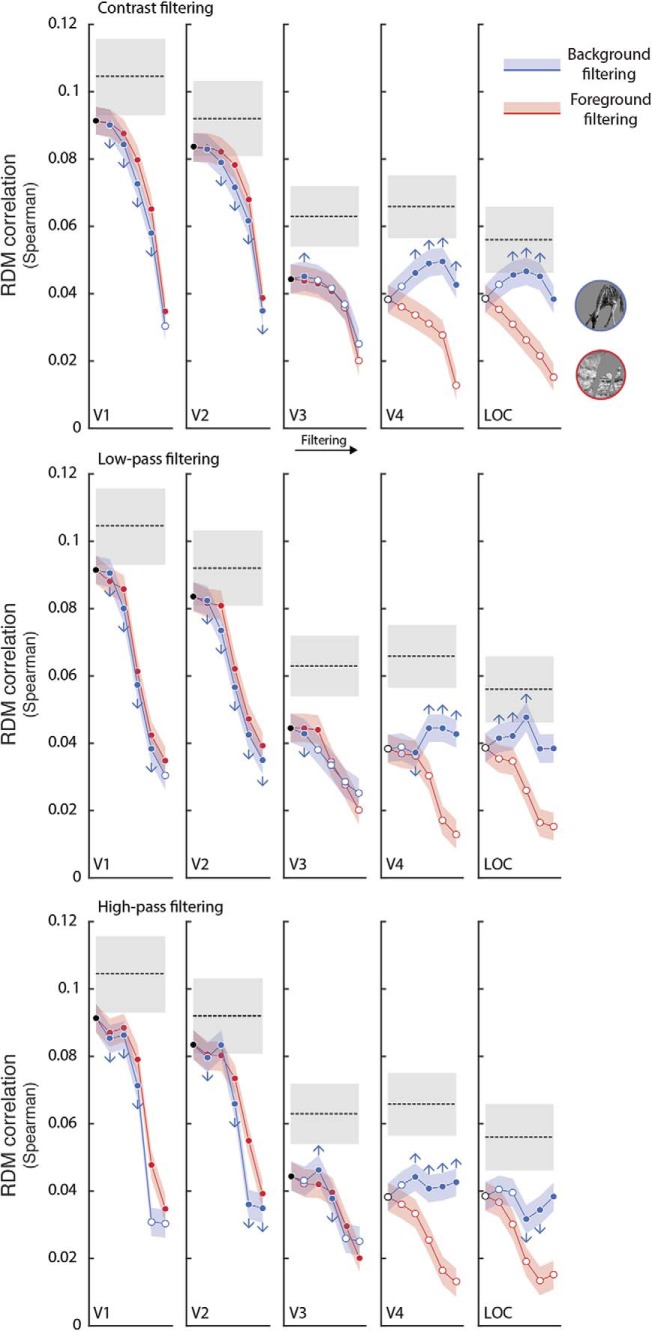
Background suppression in the human visual system. Correlation between brain activity and contrast, low- and high-pass filtering applied to the background (blue) and, as a control, to the foreground (red). Filled dots mark significant correlations (*p* < 0.05, Bonferroni corrected) while colored shaded areas represent the SE estimates. Dashed lines represent the SNR estimate for each ROI, while gray shaded regions indicate its SE. Arrows stand for significant differences (*p* < 0.05, Bonferroni corrected) between each filtering step and correlation values for the intact version (up: background suppression; down: progressive decay). Results show that for early regions (V1–V3) background-related information is relevant, since the correlation significantly decays due to filtering (*p* < 0.05, Bonferroni corrected); on the other hand, V4 and LOC show an opposite effect, suggesting that background is suppressed in those regions.

**Figure 5.**
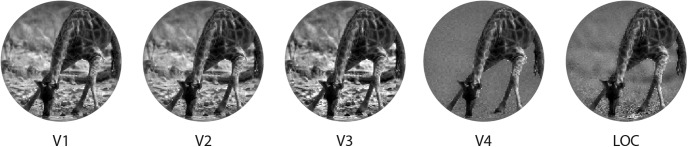
Correlation images. To visually represent these results, we combined the different filtering procedures (contrast, low- and high-pass filtering) of the step showing the highest correlation with the representational model from each ROI.

When comparing the correlation value of the intact version of the stimuli and the correlation value of each background filtering step, we found that V1, V2, and V3 show a progressive decay, indicating that the background is actually processed by these regions (*p* < 0.05, Bonferroni corrected). On the other hand, in V4 and LOC, filtering the background produces significantly higher correlations (*p* < 0.05 Bonferroni corrected), thus indicating that background information is not different from noise (Fig. 4). These findings suggest that background suppression is actually performed by higher cortical areas, as also depicted in correlation images (Fig. 5).

Of note, to validate the proposed method, we performed a simulation of the fMRI experiment using a fully connected layer of a pretrained convolutional neural network (AlexNet fc6; Krizhevsky et al., 2012). The RDM correlation between each contrast filtering step and the representational geometry from the net (i.e., responses to intact images) was computed as in the fMRI analyses (i.e., fixed model). Then, we assessed ground truth computation of the net by showing it the images at each filtering level, thus checking its sensitivity to background manipulation. Results (data not shown) demonstrate that our pre-filtering modeling approach correctly reveals the ground-truth computation of the net.

## Discussion

In the present study, we illustrated how the manipulation of low-level properties of natural images, and the following correlation with patterns of brain responses during passive viewing of the intact stimuli, could disclose the behavior of different regions along the visual pathway.

Employing this pre-filtering modeling approach, we tested whether scene segmentation is an automatic process that occurs during passive perception in naturalistic conditions, even when individuals are not required to perform any particular tasks, or to focus on any specific aspect of images. Here, we were able to collect three different pieces of evidence confirming our hypothesis on the mechanisms involved in scene segmentation.

First, by using RSA, we demonstrated that representational models built from fMRI patterns show a significant correlation with isolated foreground in V2, V4, and LOC, while a significant correlation with isolated background is achieved in V1 and V2 only.

Second, our analyses specifically found that foreground enhancement is present in all the selected visual ROIs, and that this effect is driven neither by the foreground contrast, nor by its size or location in the visual field. Thus, indirect evidence of figure-ground modulation of natural images could be retrieved in the activity of multiple areas of the visual processing stream (Roelfsema, 2006; Roelfsema and de Lange, 2016). This is consistent with a recent study, which reported that border-ownership of natural images cannot be solved by single cells but requires a population of cells in monkey V2 and V3 (Hesse and Tsao, 2016).

Finally, a proof of segmentation can be represented by the significant suppression of background-related information in V4 and LOC. On the contrary, earlier regions across the visual stream, from V1 to V3, have a uniform representation of the whole image, as evident at first glance in the obtained correlation images (Fig. 5). Overall these results further support the idea that foreground enhancement and background suppression are distinct, but associated, processes involved in scene segmentation of natural images.

### Foreground segmentation as a proxy for shape processing

Of note, our proposed pre-filtering modeling approach produces a visual representation (i.e., correlation image) of how information is selectively coded by a specific population of interest (e.g., LOC). Further interpretations on the obtained visual representation may result more empirical and, similarly to other computational neuroimaging methods (e.g., Inverted Encoding Models; Liu et al., 2018), should be grounded on previous neurophysiological knowledge. For instance, the correlation image of LOC could be interpreted as resulting from two alternative mechanisms: LOC could preferentially process the foreground as a whole, while suppressing the background, or it could act as a “feature detector,” whose neurons are selectively tuned toward a single visual attribute (e.g., the whiskers of a cat), without actively performing any suppression. Either way, what our method clearly reveals is that LOC is selective for object texture and shape properties and is unaffected by background-related information. At the same time, previous knowledge suggests that an active process, rather than a passive feature-matching mechanism, determines the observed results (Roelfsema and De Lange, 2016).

Furthermore, the observed behavior of V4 and LOC is consistent with several investigations on shape features selectivity in these regions, and in their homologues in monkey (Carlson et al., 2011; Hung et al., 2012; Lescroart and Biederman, 2013; Vernon et al., 2016). In fact, the extraction of shape properties requires segmentation (Lee et al., 1998), and presumably occurs in brain regions where background is already suppressed. As mentioned before, correlation images reconstructed from V4 and LOC are characterized by a strong background suppression, while the foreground is preserved. This is consistent with a previous neuropsychological observation: a bilateral lesion within area V4 led to longer response times in identifying overlapping figures (Leek et al., 2012). Hence, this region resulted to be crucial for accessing foreground-related computations, and presumably plays a role in matching the segmented image with stored semantic content in figure recognition. In accordance with this, a recent hypothesis suggests the role of V4 in high-level visual functions, such as features integration or contour completion (Roe et al., 2012).

The preserved spatial resolution of foreground descriptive features (i.e., texture) in V4 and LOC (as shown in Fig. 5) represents an additional noteworthy aspect that arises from our data. The progression from V1 toward higher-level regions of the cortical visual pathway is associated with a relative increase in receptive fields size (Gattass et al., 1981, 1987, 1988; Dumoulin and Wandell, 2008; Freeman and Simoncelli, 2011; Kay et al., 2015). However, it should be kept in mind that regions such as V4 demonstrate a complete representation of the contralateral visual hemifield, rather than selective responses to stimuli located above or below the horizontal meridian (Wandell and Winawer, 2011). The evidence that the foreground portion of correlation images maintains fine-grained details in V4 and LOC seems to contrast a popular view according to which these regions are more tuned to object shape (i.e., silhouettes), instead of being selective for the internal configuration of images (Malach et al., 1995; Grill-Spector et al., 1998; Moore and Engel, 2001; Stanley and Rubin, 2003). However, it has been shown that foveal and peri-foveal receptive fields of V4 do accommodate fine details of the visual field (Freeman and Simoncelli, 2011) and that the topographic representation of the central portion of this area is based on a direct sampling of the primary visual cortex retinotopic map (Motter, 2009). Therefore, given the fovea-to-periphery bias found in our stimuli and in natural images, it is reasonable that an intact configuration of the foreground may be more tied to the activity of these brain regions, and that a richer representation of the salient part may overcome simplistic models of objects shape (e.g., silhouettes). Our results are also consistent with a recent study on monkeys that demonstrates the role of V4 in texture perception (Okazawa et al., 2015).

Moreover, it is well known that selective attention represents one of the cognitive mechanisms supporting figure segmentation (Qiu et al., 2007; Poort et al., 2012), as suggested, for instance, by bistable perception phenomena (Sterzer et al., 2009), or by various neuropsychological tests (De Renzi et al., 1969; Bisiach et al., 1976). In the present experiment, participants were asked to simply gaze a central fixation point without performing any overt or covert tasks related to the presented image. Nonetheless, we found evidence of a clear background suppression and foreground enhancement, suggesting that scene segmentation is mediated by an automatic process that may be driven either by bottom-up (e.g., low-level properties of the foreground configuration), or top-down (e.g., semantic knowledge) attentional mechanisms. Neurophysiological studies suggest that segmentation is more likely a bottom-up process, as border-ownership assignment occurs as early as 70 ms (Williford and von der Heydt, 2016), followed by later region-filling mechanisms (i.e., enhancement and suppression; Self et al., 2013). A limit of our study is that we cannot provide any further information related to these mechanisms and their temporal dynamics, given the limited temporal resolution of fMRI and the passive stimulation task. However, a recent study (Neri, 2017) investigated behavioral and electrophysiological responses to BSD images, intact or manipulated in several different ways, including spatial frequencies filtering and warping, in subjects who were asked to reconstruct a corrupted image region. Results showed that reconstruction of patches elicits enhanced responses when masking targeted the behaviorally segmented contours, rather than the contrast energy of the images. Moreover, this effect occurs earlier than 100 ms and is not altered by semantic processing or spatial attention.

### Facing the challenge of explicit modeling in visual neuroscience

One of the major goals of visual neuroscience is to predict brain responses in ecological conditions (Felsen and Dan, 2005). In this sense, the standard approach in investigating visual processing implies testing the correlation of brain responses from a wide range of natural stimuli with features extracted by different alternative computational models. This approach facilitates the comparison between performances of competing models and could ultimately lead to the definition of a fully computable model of brain activity. However, the development of explicit computational models for many visual phenomena in ecological conditions is difficult. Indeed, many current theories, especially those concerning mid-level processing, have been hardly tested with natural images, as testified by the extensive use of artificial stimuli (Carandini et al., 2005; Wu et al., 2006). As a matter of fact, it is often impossible both to extract and to control for relevant features in natural images, and thus, there is no way to compute a predicted response from complex stimuli.

Moreover, even if computer vision is a major source of computational models and feature extractors, often its objectives hardly overlap with those of visual neuroscience. Computer scientists are mainly interested in solving single, distinct tasks (e.g., segmentation, recognition, etc.), while, from the neuroscientific side, the visual system is considered as a general-purpose system that could retune itself to accomplish different goals (Medathati et al., 2016). Consequently, while computer science typically employs solutions that rely only seldom on previous neuroscientific knowledge, and its goal is to maximize task accuracy (e.g., with deep learning), visual neuroscience somehow lacks of solid computational models and formal explanations, ending up with several arbitrary assumptions in modeling, especially for mid-level vision processing, such as scene segmentation or shape features extraction (for a definition, see Kubilius et al., 2014).

In light of all this, we believe that the manipulation of a wide set of natural images, and the computation of a fixed model based on low-level features, can offer a simple and biologically plausible tool to investigate brain activity related to higher-order computations, and that representational models offer an easily accountable link between brain activity and continuous stimuli descriptions (Nili et al., 2014). In fact, the results of this exploratory approach can be depicted and are as intuitive as descriptions obtained through formal modeling (Fig. 5), highlighting interpretable differences rather than data predictions.

Moreover, our study indicates that the sensitivity of representational models built on fMRI patterns can represent an adequate tool to investigate complex phenomena through the richness of natural stimuli. Representational models fit this purpose: even if are summary statistics obtained from the dissimilarities between actual brain activity, they are independent from a priori assumptions on anatomic relationships between brian regions, or on correspondences between voxels and units of computational models, as in the case of voxelwise encoding or decoding (Kriegeskorte and Kievit, 2013).

## Synthesis

Reviewing Editor: Bradley Postle, University of Wisconsin Decisions are customarily a result of the Reviewing Editor and the peer reviewers coming together and discussing their recommendations until a consensus is reached. When revisions are invited, a fact-based synthesis statement explaining their decision and outlining what is needed to prepare a revision will be listed below. The following reviewer(s) agreed to reveal their identity: Jonas Kubilius, H.Steven Scholte.

Reviewer 1: I have already reviewed this paper twice. It is refreshing to see that with every iteration the paper is improved. Overall, I find the proposed approach interesting. However, I do worry that some of the effects reported here might not reflect what the authors believe they do. I voiced those concerns in both reviews yet they have not been addressed or at least acknowledged in the present manuscript. Specifically, I ran a simulation where the ground truth computation is known and does not involve any enhancement / suppression (as I demonstrate empirically). Despite that, I was still able to replicate at least one effect reported in this manuscript. Admittedly, this is a quick and dirty simulation that is necessarily incomplete yet it casts legitimate concerns about the nature of the reported effect and needs to be addressed thoroughly.

Below I copied the majority of my original review and included some other remarks. Hopefully, these issues could be addressed in the revision of this paper.

# A thought experiment

Suppose area LOC is solely involved in object categorization. It doesn't suppress background or enhance foreground; rather, it only detects the presence of some discriminative category attributes. For concreteness, let's say we show an photo of a cat. During training, the detectors in LOC might have learned that one of the discriminative features of a cat is the whiskers. So the more confident the detector is that the whiskers are present, the stronger their activation will be.

Now whiskers are small and easy to miss in images with backgrounds. But as the background is more and more suppressed, their presence becomes easier to detect, and even simple computer vision models, such as the ones used in this study, might be able to detect those whiskers. Whiskers being more reliably detected would lead to higher correlations with LOC neural responses, but not because LOC did any background suppression.

# A simulation

This was my intuition which is a questionable source of evidence, so I also thought I could run a simulation to see if this intuition would hold in practice. First, I chose 10 objects from the same dataset and did the background filtering as described in the paper (manipulating the alpha value). Next, I simulated the fMRI experiment, showing the original images to AlexNet pre-trained on the ImageNet. So AlexNet here is the equivalent of the human brain in this paper, but unlike the brain, here the computations are known. I extracted the responses to these images at layer fc6, and computed the RDMs.

Next, I passed the images at each filtering level through GaborJet, a simple V1-like model used by Biederman that I had readily available. It's very similar to the models used in this study. The resulting RDMs were correlated with the RDM from fc6, producing plots as in Fig. 4.

As a control, I also passed the images at each filtering level through AlexNet, and got the RDMs from fc6. This is a test whether fc6 is sensitive to background manipulation. If it is, it doesn't do any figure-ground suppression (as expected from what we know about deep nets).

Here is the resulting plot: https://s29.postimg.org/lxe6mzb0n/contrast.png

First, in the plot you can see that background suppression does not happen in fc6 of AlexNet (green line, “true”). With more background suppression, the correlation went down. If fc6 did background suppression then there would be no such drop.

Next, the correlation between the simple model (GaborJet) and fc6 went up and then down, in a curiously similar fashion as seen in LOC in this paper (blue line, “model”). But no background suppression is happening in fc6!

I know that this is just a quick and dirty simulation with merely 10 images and other caveats, but due to this simulation I am concerned that the findings in this manuscript might not reflect figure-ground modulations. It would be important to validate the proposed method via a simulation like this one to make sure it gives the correct output.

# Other comments

- Computational models: In my previous review, I suggested using deep nets that have been show to provide the best correlations with human behavioral and neural data (Khaligh-Razavi & Kriegeskorte, 2014; Rajalingham et al., 2015; Kubilius et al., 2016). I can see that now the wording has been changed to “untrained models” for model selection. I don't quite see that having an “untrained” yet poorly predictive model is a good enough reason to use it. What is the motivation for using such models? I can imagine that trying out deep nets might sound like a daunting task but honestly nowadays it is really quite doable using TensorFlow (see here:https://github.com/tensorflow/models/tree/master/research/slim or here: https://keras.io/applications/) and no GPU is required.

- In the previous versions of this manuscript, error bands in Fig. 4 were significantly wider. Are they narrower now because normalization by the ceiling is no longer done?

- In the previous versions there was a Gaussian filtering experiment included as part of Fig. 4. I used the results reported for that experiment as an additional evidence that perhaps not much of figure-ground suppression is happening. What was the reason for removing this experiment? Was it flawed in some way?

Jonas Kubilius

Postdoctoral Fellow

MIT / KU Leuven

Reviewer 2: I loved reading this paper. I am currently mainly in a no reviewing mode (family circumstances) but the abstract peaked my interest. The paper did not disappoint and elegantly shows that V1 to V3 respond to background information while V4 and LOC only respond to the object, using natural stimuli.

The results are in agreement with what is expected and do not advance the field from that perspective strongly.

However, this is the first study (to my knowledge) that uses manipulated images to explore to what degree processing occurs in different areas. For me the way in which the data is analysed is very inspiring and we will see to what degree it actually extends the toolkit of computational cognitive neuroscience. It opens up investigations to the influence of attention, awareness, expectation and other visual routines within the context of a computational framework. To the degree these will be successful this paper will be a game-changer.

I do have some minor comments:

L16) abstract. There is no manipulation of tasks in Kays data so we have no idea to what degree things occur automatically or not. I give the authors it is likely the case but to conclude this in the abstract is over-extentsion.

Significance statement

L19/L27) I would add the novel approach. For me at least this is the most significant about the paper (and I like scene segmentation as a topic).

L48/L50 'The role of visual segmentation' is too broad of a formulation. Clearly ecological stimuli have been used in research into segmentation. Fair enough, the extend to which this has resulted in strong conclusions about specific mechanisms is limited, but to claim the role of visual segmentation in ecological stimuli has been ignored is incorrect.

L92. Best step? From what perspective (would help to clarify here).

Method section

The authors provide links to the data and stimuli (both I suspect from other groups). It would be nice if they would also provide github code. This will make it easier for other labs to use the novel methods introduced in the paper (and also check the results).

The authors should also provide df's with the tests (given that they are typically identical for al tests maybe in the captions).

L271 I think former should be late

Foreground is enhanced in all tested regions

It is likely my slowness but it took some time to figure out that the significant RDM correlations for figure and background are by themselves informative and not only in relation to the intact image RDM correlation. This is intrinsic to the method and might just require more reading but if the authors explain in L301/L303 that the within test is informative in itself because it is tested against alternative segmentation patterns it would have been easier to read for me.

Figure 4 and Figure 4-1

I really loved these analyses of filtering out more and more of the background. Very cook and convincing. It might be more informative if 4 and 4-1 are combined (the differences in colouring suggests this was one of the earlier stages of presentation).

Nice as this analysis is, there is no testing of a difference between the different filtering steps. Could you add this? Do the conclusions still hold?

L366. Table 2 is missing.

L410/411. These are strange references for the observation that RF size increases when going up in the hierarchy. There is a rich history before Dumoulin and Wandell, 2008, both in monkey physiology and human lesion studies.

L437. Von der Heydt and Roelfsema's labs use simple stimuli. It is entirely likely that these processes are somewhat different for simple textures than ecological stimuli with ambiguities at a more global scale. So you might conclude from Von der Heydt that segmentation for simple stimuli occurs early in time but this does not generalise.

On that matter, VAF Lamme et al., (1999, Cerebral Cortex) shows that there is both suppression of the background and enhancement of the figure in V1. Would be relevant for the intro.

L395/L448 With which realistic current view is the data not consistent?

L450/L487 I would include a reference to this in the significance statement.
